# Post-Pandemic Neutralizing Antibody Responses to SARS-CoV-2 D614G Variant in Rural and Urban Ghana

**DOI:** 10.3390/v18040414

**Published:** 2026-03-27

**Authors:** Elvis Suatey Lomotey, Irene Amoakoh Owusu, Elikem Abla Kisser, Kojo Nketia, Dorah Korkor Mensah, Angela Selase Dayi, Christopher Dorcoo, Angelica Daakyire, Peter Kojo Quashie, Irene Owusu Donkor

**Affiliations:** 1 West African Centre for Cell Biology of Infectious Pathogens, College of Basic and Applied Sciences, University of Ghana, Legon, Accra P.O. Box LG 54, Ghana; elomotey@noguchi.ug.edu.gh (E.S.L.); ireneamoakohowusu@gmail.com (I.A.O.); elikemkisser7@gmail.com (E.A.K.); dorahmensah5@gmail.com (D.K.M.); dayiangela2@gmail.com (A.S.D.); pquashie@ug.edu.gh (P.K.Q.); 2 Department of Epidemiology, Noguchi Memorial Institute for Medical Research, University of Ghana, Legon, Accra P.O. Box LG 581, Ghana; 3 Department of Parasitology, Noguchi Memorial Institute for Medical Research, University of Ghana, Legon, Accra P.O. Box LG 581, Ghana; kojonke@gmail.com (K.N.); cdorcoo@noguchi.ug.edu.gh (C.D.); adaakyire@noguchi.ug.edu.gh (A.D.)

**Keywords:** SARS-CoV-2, D614G variant, live virus assay, neutralizing antibodies

## Abstract

Africa reported lower COVID-19-related morbidity and mortality compared to other continents, despite widespread SARS-CoV-2 transmission and limited vaccine access. Proposed immunological explanations include potential pre-existing immunity such as cross-reactive humoral or cellular responses from earlier coronavirus exposures. However, functional immune responses to SARS-CoV-2 in African populations remain poorly characterized. To address this gap, we assessed post-pandemic neutralizing antibody responses against the SARS-CoV-2 D614G variant. We analyzed plasma samples from 989 participants in a cross-sectional survey in Ghana’s Eastern and Greater Accra regions. A live virus neutralization assay using Vero E6 TMPRSS2 cells was employed to quantify SARS-CoV-2 D614G-specific neutralizing antibodies. Responses were assessed across collected demographic data. Urban participants exhibited higher median neutralizing antibody titers than rural counterparts, in both vaccinated and unvaccinated groups (*p* < 0.0001). Among unvaccinated individuals, median neutralizing antibody titers were comparable across age groups in urban settings. Vaccinated individuals showed elevated median titers across all age groups, with urban residents demonstrating stronger responses. Significant sex-based differences in neutralizing titres were also identified. Our findings reveal marked disparities in functional antibody responses between urban and rural populations, likely shaped by differences in SARS-CoV-2 exposure and vaccination. Continued surveillance and immunological profiling remain key for informing vaccine strategies and future pandemic preparedness.

## 1. Introduction

Until the end of 2019, only six coronaviruses were known to infect humans. Four common, endemic viruses (OC43, HKU1, 229E and NL63) typically causing mild respiratory illness, as well as SARS-CoV and MERS-CoV, responsible for more severe disease outbreaks [1,2,3]. The emergence of a seventh, SARS-CoV-2, triggered an unprecedented pandemic [4,5]. One of the early genetic events in the pandemic was a single spike gene mutation that led to amino acid change D614G, which emerged and quickly altered the trajectory of viral spread. This mutation significantly enhanced viral infectivity and transmission efficiency, rapidly displacing the ancestral strain to become the globally dominant variant well before the emergence of subsequent variants of concern [6,7].

Despite extensive global research into anti-SARS-CoV-2 antibody responses, it remains key to understand how exposures to the virus may have influenced neutralizing antibody responses in the African population either by natural infection and/or vaccination [8,9]. While vaccination reshaped the immune landscape of anti-SARS-CoV-2 antibody responses globally, many African countries experienced delayed vaccine supply and rollout [10]. In Ghana, this delay, combined with widespread community transmission, raises important questions about the extent to which early infections, vaccine-induced responses, or hybrid immunity contribute to current SARS-CoV-2 neutralization antibody responses.

As of the time of conducting this study, Ghana had administered a total of 22,384,226 COVID-19 vaccination doses [11] equating to approximately 67 doses per 100 individuals [12]. This reflects the extent of vaccination efforts relative to the total population, which provides important context for interpreting Ghanaian population-level immune response.

This study assesses the neutralizing activity of post-pandemic plasma samples from rural and urban areas of Ghana against the D614G strain of SARS-CoV-2.

## 2. Materials and Methods

### 2.1. Study Design

This study analyzed plasma samples collected from a previously published cross-sectional survey conducted between November and December 2023 in rural and urban communities in the Eastern and Greater Accra regions of Ghana [13]. Participants were classified as residing in urban or rural areas based on Ghana Statistical Service definitions of settlements. Urban settlements (Nsawam, Medie, Amasaman, Pokuase, and Ofankor) are located along the main highway between Accra and Kumasi and are characterized by higher population density and greater access to infrastructure and services. Rural communities (Kraboa, Panpanso, Amanfrom, Doblo, and Obom) are more remote, with dispersed populations and limited access to basic infrastructure and services. Informed consent and assent were obtained from all participants prior to inclusion. Detailed demographic characteristics of the study cohort, including age and sex distributions for rural and urban participants, have been previously reported [13]. Sociodemographic characteristics, COVID-19 vaccination status, vaccine type, and biological samples were collected from 1000 participants, evenly distributed between rural and urban areas [13]. Importantly, the analyses presented in this study were conducted independently and focused specifically on live virus neutralization against the SARS-CoV-2 D614G variant.

### 2.2. Sample Collection

Whole blood (8 mL) was collected from 989 of the 1000 participants in accordance with WHO venipuncture guidelines, using BD Vacutainer^®^ CPT™ Mononuclear Cell Preparation Tubes (CPT, Becton Dickson, Franklin Lakes, NJ, USA) containing sodium citrate. CPT were transported at room temperature and promptly delivered to the laboratory at the Noguchi Memorial Institute for Medical Research (NMIMR) for further processing and analysis. Plasma was isolated via centrifugation of CPT following the manufacturer’s instructions. All samples were processed, aliquoted, and stored at −80 °C on the same day to ensure sample integrity.

### 2.3. Live Virus Neutralization Assay

**Figure 1:**
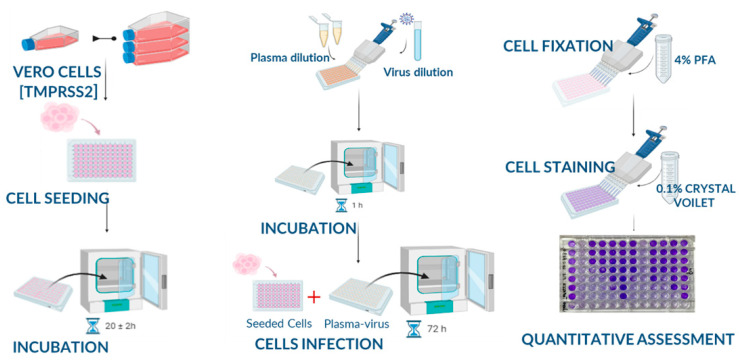
Graphical depiction of the live virus neutralization assay. Vero E6 cells were seeded, incubated, and infected with SARS-CoV-2 after plasma–virus incubation. Cells were fixed, stained with crystal violet, and assessed to determine neutralization. Created in BioRender. Owusu Donkor, I. (2026). https://BioRender.com/qncugi8.

Neutralizing activity of plasma samples against SARS-CoV-2 D614G was assessed using a crystal violet live virus endpoint assay on Vero E6/TMPRSS2 cells [14] (Figure 1). The SARS-CoV-2 virus used in the assay was isolated at the Global Immunity and Immune Sequencing for Epidemic Response (GIISER) Ghana site at the Noguchi Memorial Institute for Medical Research. The virus was obtained from a respiratory sample of a patient infected with an early pandemic SARS-CoV-2 strain carrying the D614G spike substitution, and the viral sequence was confirmed by whole-genome sequencing prior to use in the neutralization assay. Confluent cells were seeded (20,000/well, 96-well flat-bottom plates) and incubated at 37 °C, 5% CO_2_ for 20 ± 2 h. Plasma was diluted at a 1:10 ratio, followed by serial two-fold dilutions (1:20–1:1280) in 30 µL volumes. SARS-CoV-2 D614G (MOI = 0.01 TCID_50_) was added in equal volume, mixed, and incubated for 1 h at 37 °C to permit antibody–virus binding. Confluent cell monolayers were inoculated with 50 µL of the plasma–virus mixture and incubated for 72 h at 37 °C, 5% CO_2_. Cells were fixed with 4% paraformaldehyde (15 min, room temperature), stained with 0.1% crystal violet (15 min, room temperature), rinsed, and air-dried. The neutralizing titres were calculated as the reciprocal of the highest dilution completely preventing virus-induced cytopathic effect. Controls included: (i) uninfected cells only (negative control), (ii) virus-cells-only wells (positive infection control), and (iii) a plasma sample with known neutralizing activity (positive neutralizing control, (Human SARS-CoV-2 Serology Standard, Lot No. COVID-NS01097; obtained from NCI-Fredrick National Laboratory for cancer research) to ensure reliability of the assay in detecting neutralizing antibodies. Neutralization at 1:50 was used as the neutralization threshold. While many studies report neutralization at 50% inhibitory titres (NT_50_), typically above ≥1:20, this assay measures complete inhibition of cytopathic effect rather than partial neutralization. Accordingly, a conservative cut-off exceeding the minimal dilution commonly associated with non-specific effect was applied, consistent with prior reports [15,16]. 

### 2.4. Data Analysis

Neutralizing antibody titres were visualized using boxplots, across area, sex, and age group in relation to vaccination status, with the *y*-axis presented on a log2 scale. To compare differences in neutralizing antibody titres between rural and urban populations within vaccination status groups, the nonparametric Mann–Whitney U test was used for two-group comparisons. Tests were considered statistically significant with a significant level (*p*-value) set at <0.05. Figures were generated using ggplot2 in R and all analyses were performed in R statistical software (version 4.3.3).

## 3. Results

### 3.1. Significant Variation in Neutralizing Antibody Responses in Rural and Urban Cohorts

**Figure 2:**
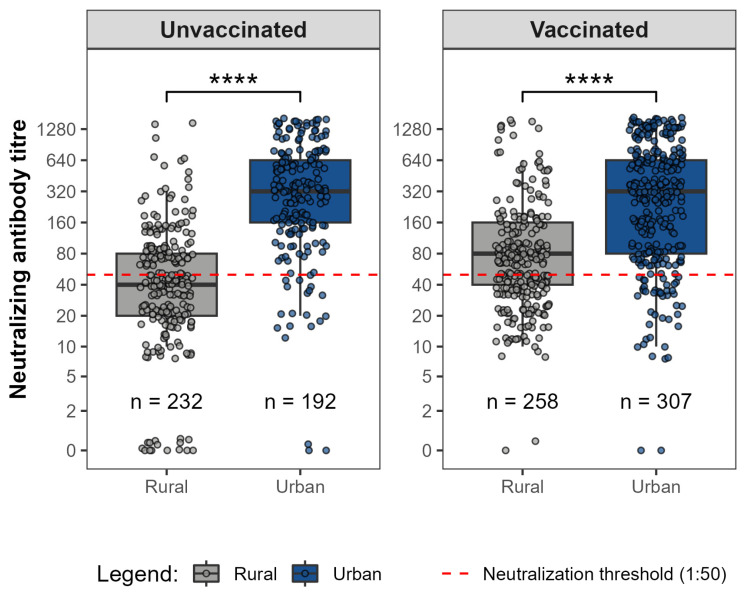
SARS-CoV-2 D614G neutralizing antibody titres among rural and urban participants by vaccination status. Boxplots show median titres and interquartile ranges, with whiskers indicating data variability. The red dashed line indicates the 1:50 neutralizing titres threshold, considered functionally relevant. Urban participants exhibited significantly higher median neutralizing titres than rural participants in both unvaccinated and vaccinated groups (**** *p* < 0.0001—Mann–Whitney U test).

Significant differences were observed in SARS-CoV-2 D614G neutralizing antibody titres between rural and urban participants. Among unvaccinated individuals, median neutralizing titres were markedly higher in urban participants compared to rural cohorts. A similar pattern was observed in the vaccinated cohort, where urban participants exhibited significantly elevated median neutralizing antibody levels relative to the rural cohort (Figure 2).

**Figure 3:**
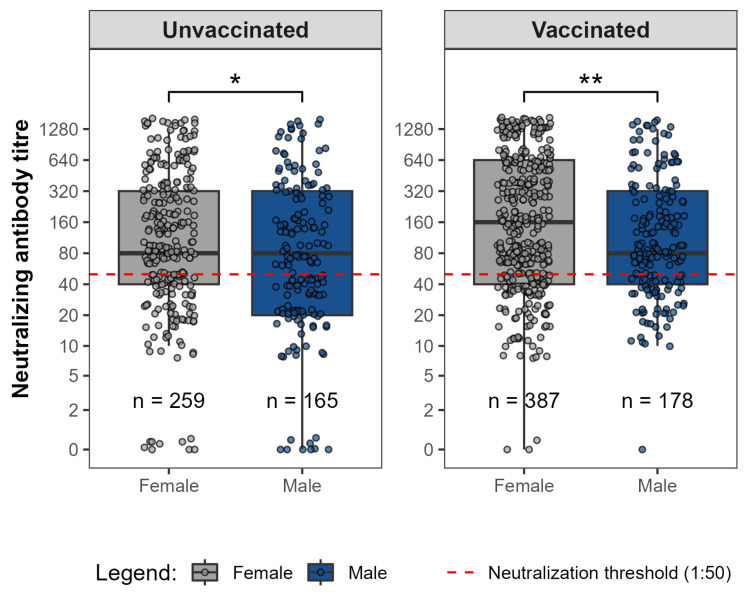
Neutralizing antibody titres against SARS-CoV-2 D614G among female and male participants stratified by vaccination status. Boxplots show median titres and interquartile ranges. The red dashed line indicates the 1:50 neutralizing titres threshold, considered functionally relevant. Statistically significant differences in neutralizing antibody titres were observed between sexes in both vaccination groups (* *p* < 0.05, ** *p* < 0.01—Mann–Whitney U test).

Neutralizing antibody titres against D614G showed overlapping distributions between female and male participants within each vaccination group (Figure 3). Among unvaccinated individuals, a statistically significant difference was observed between sexes despite similar medians and overlapping interquartile ranges. In the vaccinated group, a statistically significant sex-associated difference was also observed, with females exhibiting higher median neutralizing titres than males, despite overlapping distributions.

### 3.2. Age-Stratified Neutralizing Antibody Responses Among Individuals in Rural and Urban Ghana

**Figure 4:**
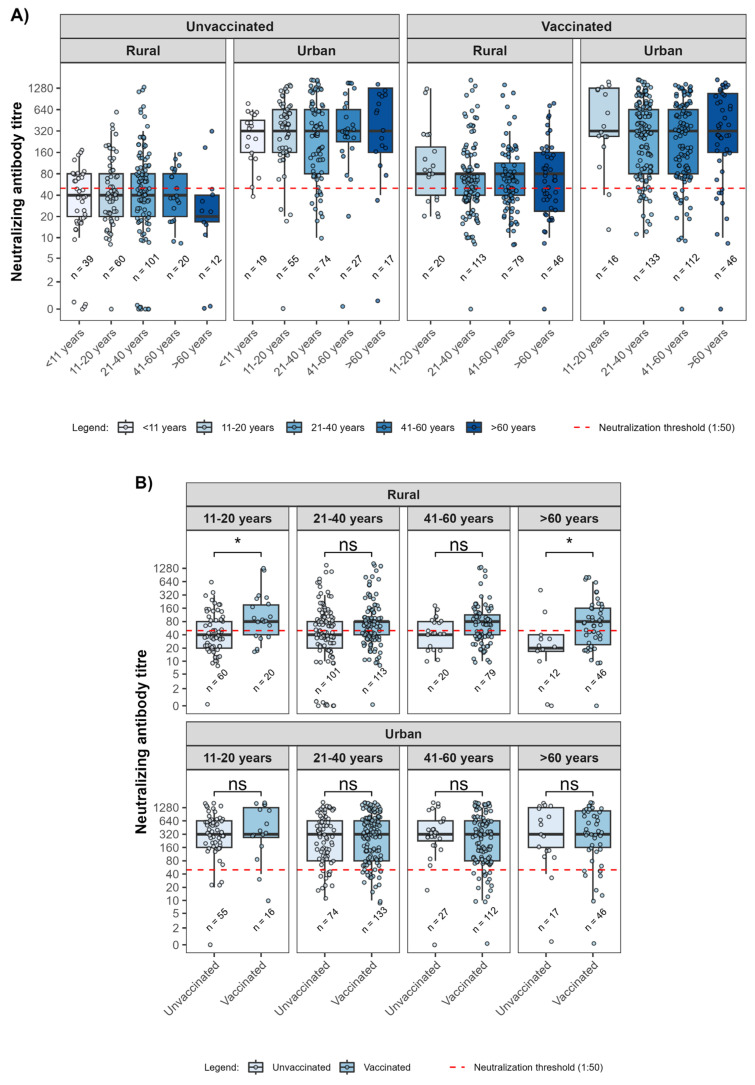
(**A**) Distribution of SARS-CoV-2 D614G neutralizing antibody titres across age groups, stratified by vaccination status and rural and urban areas. Boxplots show median titres and interquartile ranges, with whiskers indicating data variability. The red dashed line indicates the 1:50 neutralizing titres threshold, considered functionally relevant. (**B**) Comparisons of neutralizing titres between unvaccinated and vaccinated participants within each age group, stratified by rural and urban areas (ns: not significant; * *p* < 0.05 Mann–Whitney U test).

In unvaccinated individuals, median neutralizing antibody titres among urban participants were broadly comparable across age groups. In contrast, unvaccinated rural participants exhibited lower and relatively uniform titres across age groups, with medians below the neutralizing threshold. Among vaccinated participants, neutralizing titres were elevated in both rural and urban populations; however, titres remained consistently higher in urban residents. In rural vaccinated individuals, median neutralizing antibody titres exceeded the 1:50 neutralizing threshold across all age groups. Greater variability in titres was observed among older participants (Figure 4).

## 4. Discussion

This study contributes to assessing the post-pandemic landscape of neutralizing antibody responses to the SARS-CoV-2 D614G variant in Ghana, with implications for understanding immune protection dynamics. Leveraging live virus neutralization assays, a gold standard for measuring functional antibody responses [17], we show that both urban vaccinated and unvaccinated individuals exhibited higher median neutralizing antibody titres compared to their rural counterparts.

The elevated neutralizing responses among unvaccinated urban participants suggest that repeated or more intense exposure to SARS-CoV-2 likely drove the development of stronger natural immunity in these populations. This observation is consistent with epidemiological patterns in Ghana, where urban areas, particularly in Greater Accra, experienced earlier and more widespread SARS-CoV-2 transmission, contributing to the robust antibody responses detected [18]. Population density and higher interpersonal contact in these areas likely contributed to the sustained viral transmission. While previous studies have largely reported the presence of SARS-CoV-2 antibodies in these cohorts [19,20,21,22], our study advances this by assessing their functional neutralizing capacity, thereby providing a more direct measure of potential immune protection. It is also important to note that the observed population-level patterns are consistent with a previous work conducted in the same cohort, which characterized SARS-CoV-2 antibody responses using a pseudotyped virus neutralization assay. Although both studies draw from the same cross-sectional study, this study was conducted independently and was not designed as a validation or confirmatory analysis. Rather, the current findings provide complementary evidence of the population-level pattern previously observed.

Among vaccinated individuals, urban participants consistently exhibited stronger neutralizing antibody responses compared to the rural cohort. This disparity may stem from differences in vaccine cold-chain maintenance, vaccine responses, and/or vaccine types administered. However, this was not directly assessed in this study. These findings suggest targeted vaccine strategies and robust post-vaccination monitoring to ensure equitable and effective protection across all population groups.

Age-stratified analysis of unvaccinated individuals revealed no clear age-related differences in median neutralizing antibody titres. In contrast, rural unvaccinated participants showed a more uniform yet subdued antibody response across age groups, indicative of a generally lower force of infection in these settings. Among vaccinated individuals, neutralizing titres increased across all age groups; however, responses remained more variable within rural populations, potentially raising a question about the effectiveness of rural vaccinations. Interestingly, sex-based comparisons also revealed statistically significant differences in neutralizing titres with modest overlapping neutralizing antibody distributions, aligning with broader immunological evidence that sex differences in humoral immunity, while sometimes observed, may not always translate to functional neutralization [23].

Our use of a live virus neutralization assay supports the physiological relevance of our findings, particularly in evaluating functional immunity. However, this approach is resource-intensive and thus limited to the D614G variant only. While D614G remains relevant as the first globally dominant variant and a benchmark for early immune responses, it lacks key spike gene mutations found in more recent immune-evasive variants, such as those within the Omicron lineage. As such, the neutralizing activity observed may not fully represent the breadth of cross-variant protection. Therefore, extrapolating these findings to current circulating strains must be done cautiously.

## 5. Conclusions

In conclusion, our findings reveal marked variation in SARS-CoV-2 D614G neutralizing antibody responses across urban and rural populations in Ghana, driven by vaccination and possibly coronavirus exposure history. These findings stress the importance of continued surveillance with functional assays serving as a complementary tool to binding antibody measurements, especially in settings where vaccine access and pathogen exposure vary widely. As SARS-CoV-2 continues to evolve and transitions into endemicity, understanding the foundations of population-level immunity, both natural and vaccine-induced, will be key to guiding future updated or booster strategies and pandemic preparedness in Africa and beyond.

## Data Availability

All data generated or analyzed during this study are included in this published article.
